# Trends for and Clinical Factors Associated with Choice of Oral P2Y_12_ Inhibitors for Patients on Chronic Dialysis

**DOI:** 10.1007/s10557-019-06913-w

**Published:** 2019-11-15

**Authors:** Nishank Jain, Suzanne L. Hunt, Huizhong Cui, Milind A. Phadnis, Jonathan D. Mahnken, Theresa I. Shireman, Junqiang Dai, Jawahar L. Mehta, Rafia S. Rasu

**Affiliations:** 1 Department of Internal Medicine, University of Arkansas for Medical Sciences, 4301 W. Markham St, Slot 501, Little Rock, AR 72205 USA; 2 Central Arkansas Veterans Affairs Medical Center, Little Rock, AR USA; 3 Department of Biostatistics, University of Kansas School of Medicine, Kansas City, KS USA; 4 Department of Health Services, Policy and Practice, School of Public Health, Brown University, Providence, RI USA; 5 Department of Pharmacy Practice, School of Pharmacy, University of North Texas, Fort Worth, TX USA

**Keywords:** chronic dialysis,, cardiovascular,, oral P2Y12 inhibitors,, clopidogrel,, prasugrel, ticagrelor

## Abstract

**Background:**

Trends and clinical factors associated with prescribing choices for oral P2Y12 inhibitors (P2Y12-I) remain unknown for patients on chronic dialysis, i.e., with end-stage renal disease (ESRD).

**Methods:**

From 2011–2014 U.S. Renal Data System registry, we identified 36,542 ESRD patients who received new prescriptions for P2Y12-I (median age 64.0 years and 54% males). Of the cohort, 93% were receiving hemodialysis and 7% on peritoneal dialysis. We analyzed trends and investigated clinical factors associated with specific P2Y12-I prescribed.

**Results:**

Clopidogrel was prescribed for 95%, prasugrel for 3%, and ticagrelor for 2%. Clopidogrel was favored for those ≥75 years (18% of cohort). Compared to Caucasians, African Americans (36% of cohort) and Hispanics (19% of cohort) were less likely to receive prasugrel and ticagrelor (*P*<0.05). Patients receiving hemodialysis versus peritoneal dialysis were less likely to receive prasugrel over clopidogrel, adjusted odds ratio (aOR) 0.67 (0.55-0.82). Each additional year of dialysis decreased the odds of receiving prasugrel over clopidogrel, aOR 0.91 (0.85-0.98). History of atrial fibrillation reduced the odds of receiving ticagrelor or prasugrel over clopidogrel, aOR 0.69 (0.54-0.89) and 0.73 (0.60-0.89), respectively. Concomitant oral anticoagulant use was not associated with choice of P2Y12-I. Occurrence of non-ST segment elevation myocardial infarction or percutaneous coronary intervention within the 6-month period prior to the index date favored ticagrelor over prasugrel, aOR 1.31 (1.06-1.62) and 1.29 (1.01-1.66), respectively. However, prescribing trends favoring ticagrelor over prasugrel were not observed for deployment of drug-eluting, or multiple coronary stents.

**Conclusion:**

Between 2011 and 2014, clopidogrel remained the most common P2Y12-I whereas ticagrelor and prasugrel remained underutilized in ESRD patients. Prescribing practices for these drugs were based upon clinically approved indication for their use in the general population as well as perceived complexity of an ESRD patient including demographics, dialysis-related factors and comorbidities. Comparative effectiveness studies involving ESRD patients are needed to prove that ticagrelor and prasugrel are just as safe and effective as clopidogrel before clinicians can make informed decisions for choice of P2Y12-I in this patient population.

**Electronic supplementary material:**

The online version of this article (10.1007/s10557-019-06913-w) contains supplementary material, which is available to authorized users.

## Introduction

From 2011 to 2014, more than one million patients with end-stage renal disease (ESRD) on chronic dialysis in the US.^1^ Patients with ESRD, compared to the general population, are at a disproportionately higher risk of developing and of dying from thrombotic cardiovascular (CV) events,^2^ and they experience increased rates of hospitalization, adverse clinical outcomes, and higher health care-related costs. CV events can be managed with several approaches that include percutaneous coronary interventions (PCIs) and prescription medications with antiplatelet drugs. As such, oral P2Y_12_ inhibitors (P2Y12-I), such as clopidogrel, prasugrel, or ticagrelor, are antiplatelet drugs commonly prescribed for these patients and are among the top 15 medication prescribed to this patient population.^1^ Because the landmark randomized clinical trials (RCTs) of P2Y12-I systematically excluded this patient population, there is limited data to guide physicians in use of P2Y12-I in the setting of ESRD. Although recent observational study reported comparative effectiveness of ticagrelor versus clopidogrel in patients with estimated glomerular filtration rate between 15-60 ml/min,^3, 4^data remain scarce for ESRD patients. Little is known about the factors associated with physicians’ choices of prescribing P2Y12-I for this patient population as large-scale, real-world prescribing patterns of P2Y12-I have not been previously reported. Specifically, it remains unclear whether choice of P2Y12-I is determined by baseline differences in demographics, dialysis-related factors, comorbidities or differences in FDA-approved clinical indication for P2Y12-I. We used the U.S. Renal Data System (USRDS) registry data of Medicare beneficiaries with ESRD on chronic dialysis and investigated semiannual trends in P2Y12-I prescriptions for this patient population and clinical risk factors independently associated with these prescriptions. We hypothesized that demographics, dialysis-related factors and comorbidities would be associated with choice of P2Y12-I in ESRD patients.

## MATERIALS AND METHODS

### Data Source

We used data from USRDS, a national registry of patients with ESRD that includes demographic and comorbidity conditions documented upon initiation of dialysis; dialysis treatment type over time; date and cause(s) of death; and patient-level Medicare institutional (Part A), physician-supplier (Part B), and prescription drug (Part D) claims.^5^ Patient-level demographic data, clinical data, dialysis modality, and first service date of dialysis is generated upon initiation of dialysis, when nephrologists are required to submit a Medical Evidence Form (CMS-2728) to regional ESRD Networks. Each ESRD Network then forwards the information to the USRDS Coordinating Center and continues to provide regular patient updates. Medicare Part A claims include dates of admissions to hospitals, primary diagnosis, procedures performed during hospitalization, and contributing or concurrent diagnoses (based on International Classification of Diseases 9 [ICD-9-CM] codes and diagnosis-related grouping [DRG]) for each admission. Medicare Part B claims include details of outpatient services (e.g., dates of service, ICD-9-CM diagnosis, procedure codes). Medicare Part D claims include details of prescription drug use (e.g., P2Y12-I type, strength, days supply). Source files are linked with a patient-specific USRDS_ID.

### Study Design and Cohort

Institutional Review Board approvals were obtained from the University of Arkansas for Medical Sciences, Little Rock, AR, and University of Kansas Medical Center (KUMC), Kansas City, KS (primary site for data collection, processing, and analysis). Subsequently, a data-use agreement was signed and approved by the USRDS Program Director.

We created a retrospective national cohort of prevalent ESRD patients from USRDS registry data between July 20, 2011, and December 31, 2014. The start date was chosen because it is the date when ticagrelor became available in the market, which allowed us to create a contemporary ESRD cohort and limit bias in the results by focusing on a period when all three P2Y12-I were available. The end date was, at the time of analyses, the latest date for which USRDS had released data. After applying exclusions, we identified every continuously eligible patient in the dataset who was given a new prescription for a P2Y12-I. Two steps were used to identify new prescriptions. First, prescriptions for P2Y12-I were identified from nonproprietary drug names in Medicare Part D claims. Second, any prescription that appeared after a 6-month period with no P2Y12-I exposure was assumed to be a new prescription. Because pharmacologic effects of P2Y12-I occur and wash-out relatively quickly, 6 months without exposure seemed suitable.

**Figure 1:**
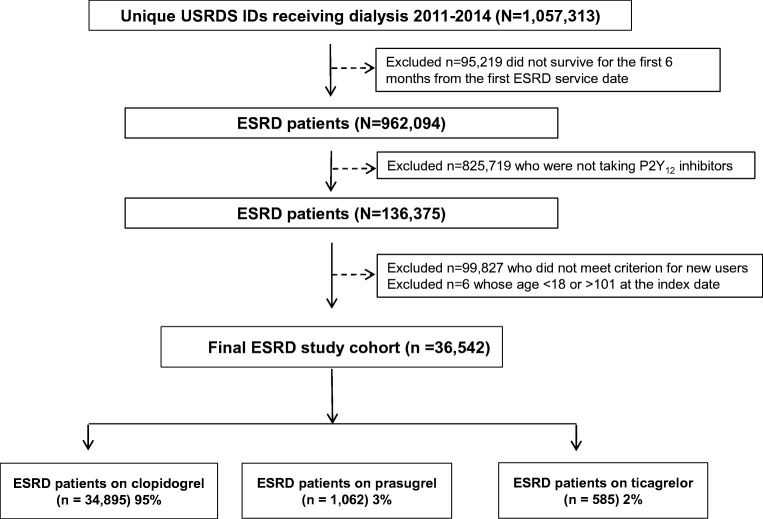
Derivation of the study cohort. From 1,057,313 unique U.S. Renal Data System (USRDS) identification numbers (IDs), we applied exclusion criteria to arrive at the final cohort of 36,542 patients with end stage renal disease (ESRD) who were on chronic dialysis and received new prescriptions for oral P2Y_12_ inhibitors.

The study cohort included patients undergoing hemodialysis or peritoneal dialysis (detailed in Figure 1). Those who received transplants but returned to dialysis because of failed allografts before index dates were considered for inclusion. We included any patient who was at least 18 years of age; was receiving chronic maintenance dialysis; had survived at least 6 months from first USRDS-recorded service; was continuously eligible for Medicare Parts A, B, and D 180 days before the index date; and received new prescriptions for P2Y12-I. A patient was excluded if younger than 18 years of age; date of first USRDS-recorded service was missing; dialysis started after the study end date; eligibility for Medicare Parts A, B, and/or D was noncontinuous; chronic maintenance dialysis treatments were not received; P2Y12-I was not prescribed; or P2Y12-I prescription was not new. When two different P2Y12-I were prescribed on the index date (N=3), the P2Y12-I observed in the subsequent prescription was considered the index drug.

### Variables

The primary outcome was a new prescription for a P2Y12-I, which reduced the impact of prevalence bias.^6^ The index date was defined as date of the new P2Y12-I prescription. The earliest possible index date was July 20, 2011, when ticagrelor became available. Demographics data was collected from CMS-2728 (recorded at first USRDS service). Information related to dialysis treatment, including modality (hemodialysis versus peritoneal dialysis), vintage (time between dialysis initiation and index date), and underlying cause of ESRD, was collected. Medicare enrollment files in USRDS registry data were used to identify continuous Parts A, B and D eligibility and enrollment in low-income subsidy. Comorbidities were collected from CMS-2728 and combined with codes appearing on 2 different days in outpatient claims data or once in hospital claims data 6 months before the index date. Details of ICD-9, diagnosis related group (DRG), current procedural terminology (CPT), and healthcare common procedure coding system (HCPCS) codes that were used to identify comorbidities are provided in Supplementary Table 1. Modified Liu comorbidity index, a validated measure of comorbidity burden in ESRD patient population, was calculated using algorithm previously published (Supplementary Table 1).^7^

### Statistical Analyses

Summary data on the number of patients receiving P2Y12-I prescriptions was reported. A Cochran Armitage trend test was performed to determine linear trends in P2Y12-I use. Descriptive statistics were generated for continuous variables stratified by P2Y12-I, and nonparametric Kruskal-Wallis test was used to test for statistically significant differences. Multiplicity adjustments for post hoc pairwise comparisons between the groups were made with the Dwass-Steel-Critchlow-Fligner test when the overall model F-test showed statistical significance.^8^ Categorical variables were compared with the Cochran-Mantel-Haenszel chi-square tests. Independent associations between baseline characteristics (e.g., demographics, dialysis-related variables and comorbidities) and P2Y12-I prescriptions were analyzed with multivariable polychotomous logistic regression. In the multivariable analyses, we modeled the odds of receiving a P2Y12-I prescription compared to another P2Y12-I prescription (e.g., ticagrelor versus clopidogrel) as an independent function of baseline characteristics (i.e., demographics, dialysis-related or comorbidity). Clinically relevant or statistically significant baseline characteristics in univariable analyses were included in multivariable analyses. All statistical tests were conducted at the 5% level of significance. Analyses were generated with SAS software, Version 9.4 of the SAS System for Windows (SAS Institute Inc., Cary, NC).

## RESULTS

USRDS records for 2011 through 2014 included 962,094 patients whose date of first service was known and who had received outpatient chronic maintenance dialysis and survived 6 months from the first service date; of those, 136,375 (14.2%) received P2Y12-I prescriptions. After applying the criterion for new P2Y12-I prescriptions, we selected the final cohort of 36,542 (3.8%) patients with ESRD.

**Figure 2:**
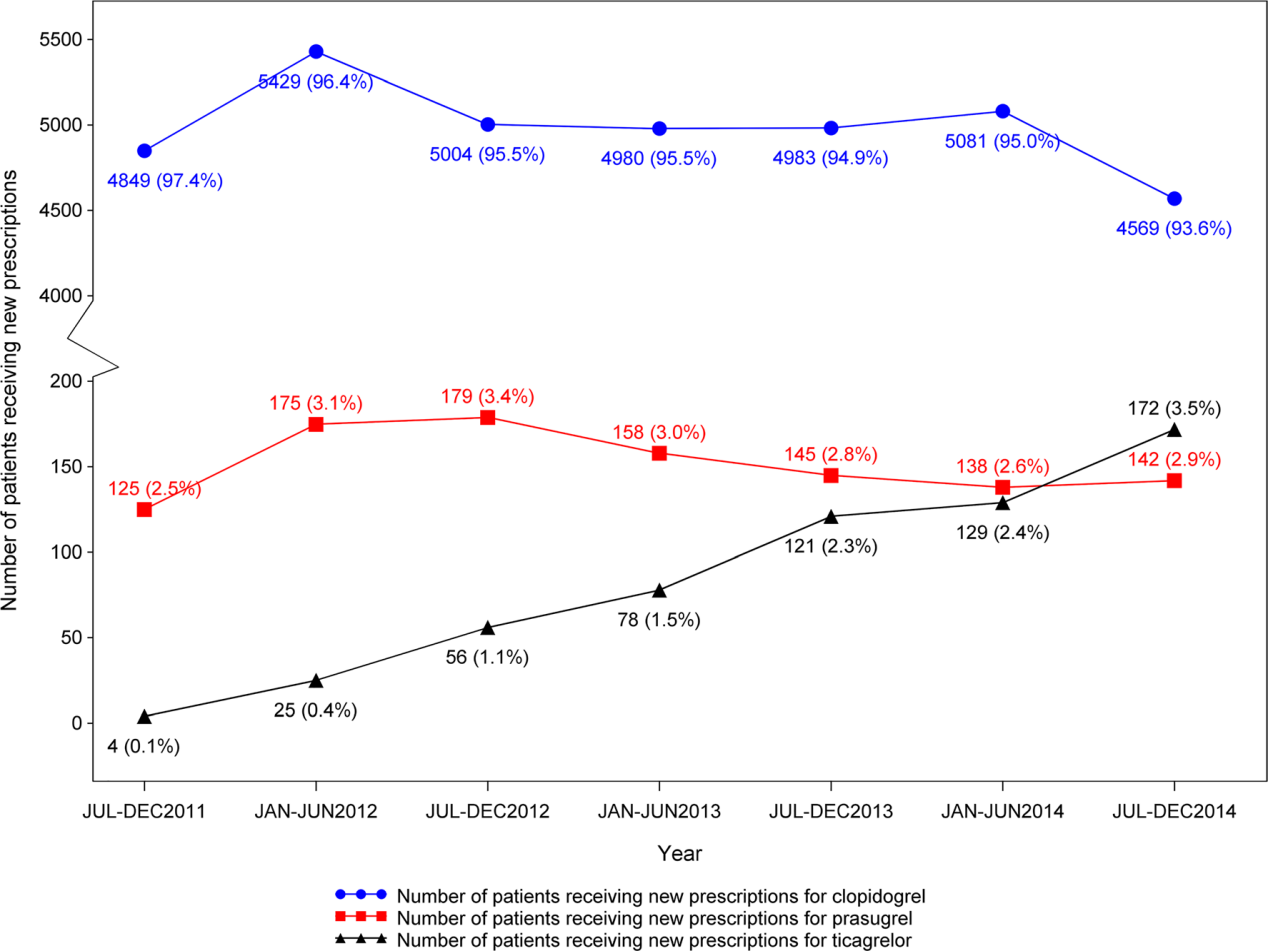
Line graph showing number of patients with end-stage renal disease on chronic dialysis who received new prescriptions for oral P2Y_12_ inhibitors (Y-axis) over 6-month intervals from July 2011 (when ticagrelor first became available in the market) through December 2014 (end of study period). Blue circles, patients receiving clopidogrel; red squares, patients receiving prasugrel; black triangles, patients receiving ticagrelor. *P*-for trend <0.0001, 0.61 and <0.0001, respectively.

Of the 36,542 ESRD patients who received new P2Y12-I prescriptions, 95% (n = 34,895) received clopidogrel, 3% received prasugrel (n = 1,062), and 2% received ticagrelor (n = 585) (Figure 2). There was an increasing linear trend (*P*<0.0001) in the proportion of patients receiving ticagrelor from July 2011 (0.1%) to December 2014 (3.5%). Likewise, a decreasing linear trend (*P*<0.0001) was observed in the proportion of patients receiving clopidogrel from July 2011 (97.4%) to December 2014 (93.6%). No significant linear trend (*P*=0.61) was observed for patients receiving prasugrel during this period.

Table 1 shows demographics, dialysis-related factors and comorbidities of the cohort. Median (IQR) age of the cohort was 64.0 years (55.0, 72.0) and 17.7% were 75 years or older. The cohort comprised of 54% men, 36% African American, and 18% Hispanic. Of the cohort, 93% were on hemodialysis and the remaining on peritoneal dialysis. Nearly 20% of the cohort had atrial fibrillation and less than 9% were prescribed oral anticoagulants on the index date. During the 6-month period prior to the index date, 28% of the cohort underwent PCI, 23% had acute myocardial infarction (AMI), 8% encountered non-fatal cardiogenic shock or cardiac arrest and 2% had coronary artery bypass graft (CABG) (Table 2).

**Table 1:** Baseline characteristics of the cohort: Demographics, Dialysis-Related Factors and Major Comorbidities

Baseline Characteristics	All	clopidogrel	prasugrel	ticagrelor	p-value^1^
Age^2^, median(IQR)	64.0 (55.0, 72.0)	64.0 (55.0, 73.0)	60.0 (52.0, 68.0)	64.0 (56.0, 72.0)	<0.0001
Categories of Age, n(%)					
18-64	18,605 (50.9%)	17,620 (50.5%)	691 (65.1%)	294 (50.3%)	
65-75	11,478 (31.4%)	10,974 (31.4%)	310 (29.2%)	194 (33.2%)	
> 75	6,459 (17.7%)	6,301 (18.1%)	61 (5.7%)	97 (16.6%)	
Gender: Male, n(%)	19,633 (53.7%)	18,667 (53.5%)	645 (60.7%)	321 (54.9%)	<0.0001
Ethnicity, Hispanic / Latino, n(%)	6,950 (19.0%)	6,640 (19.0%)	220 (20.7%)	90 (15.4%)	
Race, n(%)					<0.0001
African American, Black	13,200 (36.1%)	12,753 (36.5%)	282 (26.6%)	165 (28.2%)	
Caucasian	14,680 (40.2%)	13,895 (39.8%)	490 (46.1%)	295 (50.4%)	
Hispanic, White	6,643 (18.2%)	6,338 (18.2%)	218 (20.5%)	87 (14.9%)	
Other Race	1,959 (5.4%)	1,851 (5.3%)	71 (6.7%)	37 (6.3%)	
Residence Location, n(%)					0.0571
Metropolitan ≥1million	19,453 (53.2%)	18,618 (53.4%)	537 (50.6%)	298 (50.9%)	
Metro <1million or adjacent to Metro	14,992 (41.0%)	14,271 (40.9%)	473 (44.5%)	248 (42.4%)	
Rural/Urban (not adjacent to Metro)	2,034 (5.6%)	1,943 (5.6%)	52 (4.9%)	39 (6.7%)	
Low Income Subsidy at Any Time, n(%)	26,314 (72.0%)	25,178 (72.2%)	751 (70.7%)	385 (65.8%)	0.0020
Etiology of ESRD, n(%)					0.0236
Diabetes	20,440 (55.9%)	19,471 (55.8%)	628 (59.1%)	341 (58.3%)	
Glomerulonephritis	2,610 (7.1%)	2,480 (7.1%)	90 (8.5%)	40 (6.8%)	
Hypertension	9,076 (24.8%)	8,724 (25.0%)	218 (20.5%)	134 (22.9%)	
Other	4,157 (11.4%)	3,971 (11.4%)	120 (11.3%)	66 (11.3%)	
Dialysis Modality, n(%)					<0.0001
Hemodialysis	34,066 (93.2%)	32,593 (93.4%)	945 (89.0%)	528 (90.3%)	
Peritoneal Dialysis	2,476 (6.8%)	2,302 (6.6%)	117 (11.0%)	57 (9.7%)	
Dialysis vintage (years), median(IQR)	3.8 (1.9, 6.6)	3.8 (1.9, 6.7)	3.5 (1.7, 6.2)	3.5 (1.7, 6.0)	0.0037
Anticoagulant use at index date, n(%)	3,195 (8.7%)	3,050 (8.7%)	93 (8.8%)	52 (8.9%)	0.9920
Major Comorbidities					
Hypertension, n(%)	32,783 (89.7%)	31,309 (89.7%)	952 (89.6%)	522 (89.2%)	0.8690
Diabetes, n(%)	28,889 (79.1%)	27,562 (79.0%)	848 (79.8%)	479 (81.9%)	0.1895
Cancer, n(%)	3,303 (9.0%)	3,170 (9.1%)	74 (7.0%)	59 (10.1%)	0.2747
Liver disease, n(%)	2,929 (8.0%)	2,810 (8.1%)	84 (7.9%)	35 (6.0%)	0.1864
Gastrointestinal bleeding, n(%)	3,555 (9.7%)	3,413 (9.8%)	88 (8.3%)	54 (9.2%)	0.2480
COPD, n(%)	11,434 (31.3%)	10,982 (31.5%)	284 (26.7%)	168 (28.7%)	0.0019
Peripheral vascular disease, n (%)	15,514 (42.5%)	17,879 (51.2%)	422 (39.7%)	244 (41.7%)	<0.0001
Atrial fibrillation, n(%)	7,184 (19.7%)	6,923 (19.8%)	160 (15.1%)	101 (17.3%)	0.0002
Congestive heart failure, n(%)	22,642 (62.0%)	21,597 (61.9%)	674 (63.5%)	371 (63.4%)	0.4452
Ischemic stroke, n(%)	4,584 (12.5%)	4,511 (12.9%)	38 (3.6%)	35 (6.0%)	<0.0001
Intracranial hemorrhage, n(%)	363 (1.0%)	355 (1.0%)	4 (0.4%)	4 (0.7%)	0.0871
Modified Liu Index (180), median(IQR)	7.0 (4.0, 11.0)	7.0 (4.0, 11.0)	7.0 (4.0, 10.0)	7.0 (4.0, 10.0)	0.0146
Categories, modified Liu Index (180), n (%)					
0	973 (2.7%)	956 (2.7%)	10 (0.9%)	7 (1.2%)	
1-4	9,313 (25.5%)	8,851 (25.4%)	319 (30.0%)	143 (24.4%)	
5-8	11,387 (31.2%)	10,852 (31.1%)	328 (30.9%)	207 (35.4%)	
9-12	10,238 (28.0%)	9,763 (28.0%)	313 (29.5%)	162 (27.7%)	
13+	4,631 (12.7%)	4,473 (12.8%)	92 (8.7%)	66 (11.3%)	

^1^p-values were calculated by Kruskal-Wallis test (continuous variables) and chi-square test (categorical variables). ^2^Ages were calculated on index date.

**Table 2:** Cardiovascular Events of the Cohort During the 6-Month Period Prior to the Index Date

Cardiovascular events	Overall	Clopidogrel	Prasugrel	Ticagrelor	p-value
Acute myocardial infarction, n (%)	8,418 (23.0%)	7,749 (22.2%)	403 (37.9%)	266 (45.5%)	<0.0001
STEMI, n (%)	785 (2.1%)	709 (2.0%)	51 (4.8%)	25 (4.3%)	<0.0001
NSTEMI, n (%)	6,974 (19.1%)	6,440 (18.5%)	322 (30.3%)	212 (36.2%)	<0.0001
Non-fatal cardiogenic shock or cardiac arrest, n (%)	2,937 (8.0%)	2,781 (8.0%)	103 (9.7%)	53 (9.1%)	0.0817
Percutaneous coronary intervention, n(%)	11,978 (32.8%)	10,655 (30.5%)	837 (78.8%)	486 (83.1%)	<0.0001
Multiple coronary stents	5,663 (15.5%)	4,937 (14.1%)	465 (43.8%)	261 (44.6%)	<0.0001
Drug-eluting stent	5,478 (15.0%)	4,702 (13.5%)	490 (46.1%)	286 (48.9%)	<0.0001
Bare-metal stent	10,366 (28.4%)	9,219 (26.4%)	746 (70.2%)	401 (68.5%)	<0.0001
Coronary artery bypass graft, n (%)	862 (2.4%)	850 (2.4%)	7 (0.7%)	5 (0.9%)	<0.0001

Multivariable associations of demographics, dialysis-related factors and comorbidities with choice of a P2Y12-I prescription over another are presented (Table 3). Age was independently associated with the choice of P2Y12-I—for every 10-year increase in age, the odds of receiving ticagrelor over prasugrel were 27% higher (*P*<0.0001), and of receiving prasugrel over clopidogrel were 24% lower (*P*<0.0001). Compared to Caucasians, the odds of receiving ticagrelor over clopidogrel was 36% lower for African Americans (*P*<0.0001) and 32% lower for Hispanics (*P*=0.003). There was lower odds for African Americans to receive prasugrel over clopidogrel (*P*<0.0001). Receiving low income subsidy was not associated with choice of P2Y12-I in the multivariable model (Table 3). Patients on hemodialysis compared to peritoneal dialysis had 33% lower odds of receiving prasugrel over clopidogrel (*P*=0.0001). For every 1-year increase in time on dialysis, there was 9% lower odds of receiving prasugrel over clopidogrel (*P*=0.0165). Etiology of ESRD was not associated with choice of P2Y12-I prescription (Table 3). Presence of comorbidities such as atrial fibrillation, chronic obstructive pulmonary disease (COPD), ischemic stroke or peripheral vascular disease (PVD) reduced odds of receiving newer P2Y12-I over clopidogrel (*P*<0.01). Concurrent anticoagulant use at index date was not associated with choice of P2Y12-I. Finally, year of index date was significantly associated with choice of P2Y12-I- favoring ticagrelor over other P2Y12-I (*P*<0.0001) and no difference in receiving prasugrel over clopidogrel (Table 3).

**Table 3:** Multivariable Polychotomous Regression Model to Investigate Association Between Choice of an Oral P2Y_12_ Inhibitor and Baseline Characteristics (Demographics, Dialysis-Related and Major Comorbidities)

Baseline characteristics	Ticagrelor vs Clopidogrel		Ticagrelor vs Prasugrel		Prasugrel vs Clopidogrel	
	OR [95% CI]	p-value	OR [95% CI]	p-value	OR [95% CI]	p-value
Age at index date, per 10 years	0.96 [0.90 – 1.04]	0.3154	1.27 [1.16 – 1.39]	<0.0001	0.76 [0.72 – 0.81]	<0.0001
Male gender	0.99[0.84 – 1.18]	0.9464	0.81 [0.65 – 0.99]	0.0425	1.23[1.09 – 1.4]	0.0013
Race, Caucasian as reference						
African American	0.64 [0.52 – 0.79]	<0.0001	1.02 [0.79 – 1.32]	0.8753	0.63 [0.53 – 0.74]	<0.0001
Hispanic	0.68 [0.52 – 0.79]	0.0027	0.71 [0.52 – 0.96]	0.0261	0.96 [0.80 – 1.14]	0.6122
Others	0.93 [0.66 – 1.33]	0.6989	0.89 [0.58 – 1.37]	0.6068	1.04 [0.81 – 1.35]	0.7416
Hemodialysis (versus peritoneal dialysis)	0.76 [0.57 – 1.01]	0.0607	1.14 [0.81 – 1.61]	0.4577	0.67 [0.55 – 0.82]	0.0001
Ln(dialysis vintage in days), per unit increase	0.96 [0.87 – 1.06]	0.3760	1.05 [0.93 – 1.18]	0.4569	0.91 [0.85 – 0.98]	0.0165
Etiology of ESRD, diabetes as reference						
Glomerulonephritis	0.88 [0.63 – 1.24]	0.4649	0.92 [0.61 – 1.39]	0.7071	0.95 [0.75 – 1.20]	0.6841
Hypertension	0.89 [0.72 – 1.09]	0.2670	1.04 [0.80 – 1.35]	0.7745	0.86 [0.73 – 1.01]	0.0586
Others	0.84 [0.64 – 1.11]	0.2122	0.98 [0.70 – 1.38]	0.9078	0.86 [0.70 – 1.05]	0.1404
Year of index date						
2012 versus 2011	9.43 [3.45 – 25.76]	<0.0001	7.10 [2.55 – 19.78]	0.0002	1.33 [1.08 – 1.64]	0.0075
2013 versus 2012	2.54 [1.95 – 3.29]	<0.0001	2.87 [2.13 – 3.88]	<0.0001	0.88 [0.75 – 1.03]	0.1173
2014 versus 2013	1.56 [1.30 – 1.88]	<0.0001	1.63 [1.28 – 2.08]	<0.0001	0.96 [0.81 – 1.13]	0.6257
Receiving low income subsidy at any time	0.84 [0.20 – 3.52]	0.8166	0.48 [0.04 – 5.41]	0.5555	1.75 [0.24 – 12.74]	0.5823
Continuous eligibility to Medicare Parts A, B, D and without receiving low income subsidy or Medicare Advantage plan	0.96 [0.23 – 4.00]	0.9539	0.44 [0.04 – 4.93]	0.5055	2.18 [0.30 – 15.9]	0.4426
Oral anticoagulant use at index date	1.09 [0.80 – 1.48]	0.5882	0.95 [0.65 – 1.38]	0.7824	1.15 [0.91 – 1.45]	0.2390
History of chronic obstructive pulmonary disease	0.75 [0.61 – 0.93]	0.0076	0.97 [0.75 – 1.26]	0.8168	0.78 [0.66 – 0.91]	0.0017
History of atrial fibrillation	0.69 [0.54 – 0.89]	0.0035	0.94 [0.69 – 1.29]	0.7092	0.73 [0.60 – 0.89]	0.0017
History of ischemic stroke	0.39 [0.27 – 0.55]	<0.0001	1.61 [0.99 – 2.61]	0.0504	0.24 [0.17 – 0.33]	<0.0001
History of peripheral vascular disease	0.59 [0.49 – 0.71]	<0.0001	1.03 [0.82 – 1.29]	0.8054	0.57 [0.50 – 0.66]	<0.0001

Abbreviation: CI-confidence interval, ESRD-end stage renal disease, OR-odds ratio

Multivariable associations of cardiac events occurring within the 6-month period prior to the index date with choice of a P2Y12-I prescription over another are presented (Table 4). In the setting of NSTEMI and PCI compared to absence of such events, there were higher odds that a patient would receive a prescription for ticagrelor over other P2Y12-I even after adjusting for confounders in various hierarchical regression models (Table 4). For placement of drug-eluting coronary stents or multiple coronary stents- there were higher odds that a patient would receive a prescription for ticagrelor or prasugrel over clopidogrel and no difference in the odds of receiving ticagrelor over prasugrel (Table 4). In the setting of non-fatal cardiogenic shock or non-fatal cardiac arrest, there were higher odds of receiving prasugrel over clopidogrel (Table 4).

**Table 4:** Multivariable Polychotomous Regression Model* Analyzing Associations Between Choice of an Oral P2Y_12_ Inhibitor and Cardiac Events Occurring During the 6-Month Period Prior to the Index Date of a Prescription

Covariates	Pairwise Drug comparison	Univariable		Plus Demographics		Plus Dialysis-related factors		Plus Comorbidities	
		OR [95%CI]	p-value	OR [95%CI]	p-value	OR [95%CI]	p-value	OR [95%CI]	p-value
NSTEMI	T vs C	2.51 [2.11 – 2.98]	<0.0001	2.46 [2.07 – 2.92]	<0.0001	2.47[2.08– 2.93]	<0.0001	2.31 [1.93 – 2.77]	<0.0001
	T vs P	1.31 [1.06 – 1.62]	0.0142	1.25 [1.01 – 1.55]	0.0394	1.25 [1.01 – 1.55]	0.0416	1.23 [0.98 – 1.54]	0.0737
	P vs C	1.92 [1.68 – 2.20]	<0.0001	1.96 [1.72 – 2.24]	<0.0001	1.98[1.73 – 2.26]	<0.0001	1.88 [1.63 – 2.17]	<0.0001
PCI	T vs C	12.93 [10.48 – 15.94]	<0.0001	12.68 [10.28 – 15.65]	<0.0001	12.64 [10.25 – 15.60]	<0.0001	13.02 [10.54 – 16.10]	<0.0001
	T vs P	1.29 [1.01 – 1.66]	0.0492	1.31 [1.01 – 1.68]	0.0394	1.31 [1.02 – 1.69]	0.0380	1.37 [1.05 – 1.77]	0.0195
	P vs C	10.03 [8.67 – 11.60]	<0.0001	9.71 [8.39 – 11.24]	<0.0001	9.66 [8.35 – 11.19]	<0.0001	9.96 [8.60 – 11.54]	<0.0001
Multiple stent	T vs C	4.89 [4.14 – 5.77]	<0.0001	4.78 [4.05 – 5.64]	<0.0001	4.79 [4.06 – 5.33]	<0.0001	4.97 [4.18 – 5.90]	<0.0001
	T vs P	1.03 [0.84 – 1.27]	0.7454	1.02 [0.83 – 1.25]	0.8384	1.02 [0.83 – 1.25]	0.8529	1.09 [0.89 – 1.35]	0.4002
	P vs C	4.73 [4.17 – 5.37]	<0.0001	4.68 [4.13 – 5.31]	<0.0001	4.70 [4.14 – 5.33]	<0.0001	4.54 [3.98 – 5.17]	<0.0001
Drug eluting stent	T vs C	6.14 [5.21 – 7.24]	<0.0001	5.99 [5.08 – 7.07]	<0.0001	5.99 [5.08 – 7.07]	<0.0001	5.62 [4.74 – 6.67]	<0.0001
	T vs P	1.12 [0.91 – 1.37]	0.2848	1.12 [0.91 – 1.37]	0.2849	1.12 [0.91 – 1.37]	0.2856	1.13 [0.92 – 1.39]	0.2514
	P vs C	5.50 [4.86 – 7.24]	<0.0001	5.44 [4.80 – 6.17]	<0.0001	5.37 [4.73 – 6.08]	<0.0001	4.97 [4.37 – 5.66]	<0.0001
Non-fatal cardiogenic shock or arrest	T vs C	1.15 [0.87 – 1.53]	0.3352	1.14 [0.86 – 1.52]	0.3675	1.16 [0.87 – 1.54]	0.3200	1.12 [0.83 – 1.51]	0.4615
	T vs P	0.93 [0.66 – 1.31]	0.6718	0.88 [0.62 – 1.25]	0.4773	0.88 [0.62 – 1.24]	0.4616	0.88 [0.61 – 1.26]	0.4833
	P vs C	1.24 [1.00 – 1.53]	0.0414	1.29 [1.05 – 1.59]	0.0152	1.32 [1.08 – 1.61]	0.0094	1.28 [1.02 – 1.59]	0.0297

**Abbreviations**: C-clopidogrel, P-prasugrel, T-ticagrelor, NSTEMI-non-ST segment elevation myocardial infarction, PCI-percutaneous coronary intervention

Following covariates were included in the sequential models:-

Plus demographics includes age, gender, and race.

Plus dialysis-related factors includes demographics + dialysis modality + log(dialysis vintage) in days.

Plus comorbidities includes dialysis related factors + Year of index date + History of COPD + History of atrial fibrillation + History of ischemic stroke + History of peripheral vascular disease + Modified Liu Comorbidity index.

*Sequential adjustments made for statistically or clinically significant baseline characteristics from Tables 1, 2, 3

## DISCUSSION/CONCLUSION

Using national registry data of ESRD patients on chronic dialysis between July 2011 and December 2014, we report prescribing patterns of clopidogrel, prasugrel, and ticagrelor in this understudied patient population. Clopidogrel remained the most common P2Y12-I prescribed to patients on chronic dialysis whereas ticagrelor and prasugrel remained underutilized. Age and racial differences determined choice of P2Y12-I prescriptions: clopidogrel the most favored and prasugrel the least favored with older age; and, minorities less likely to receive ticagrelor over other P2Y12-I. Among dialysis-related factors, patients receiving hemodialysis *versus* peritoneal dialysis or with every year increase in time on dialysis (i.e., *dialysis vintage*) had lower odds to receive prasugrel over clopidogrel. Presence of atrial fibrillation and COPD compared to absence of these comorbidities reduced odds of receiving ticagrelor or prasugrel over clopidogrel. Occurrence of NSTEMI or PCI compared to lack of these events within the 6-month period prior to the index date favored ticagrelor over other P2Y12-I.

Among ESRD patients on chronic dialysis in the U.S.A. between 2011-2014, clopidogrel remained the most commonly prescribed P2Y12-I, utilization of prasugrel (from 2.5% to 2.9%) remained unchanged and ticagrelor use (from 0.1% to 3.5%) was on the rise, a trend similar to that in the general population and Veterans.^9–11^ This trend is reflected by the fact that year of index date for prescription was independently associated with choice of P2Y12-I favoring ticagrelor over others in the recent years (2014 versus 2011). These trends follow the publication of the Platelet Inhibition and Patient Outcomes (PLATO) trial, which reported ticagrelor to be 23% more effective, adjusted hazard ratio 0.77 (0.65-0.90) over clopidogrel in reducing the composite outcome of cardiovascular death, AMI, or stroke at 12 months among participants with estimated glomerular filtration rate (eGFR) of <60 ml/min/1.73m^2^ (n = 2,562).^12^ This landmark trial excluded ESRD patients. There are no data for use of newer P2Y12-I in ESRD patients, and post-marketing data remains scarce on effectiveness and safety of ticagrelor or prasugrel over clopidogrel in this patient population.^13, 14^ These trends need to be monitored carefully specifically in light of the PLATO trial efficacy and safety data on the subgroup of participants with kidney disease that was published by the Food and Drug Administration (FDA) raising concerns regarding efficacy and safety of these drugs in patients with advanced kidney disease.^15^

Among the demographics variables, we found age, gender and race to be associated with choice of P2Y12-I in patients on chronic dialysis. It appears that clopidogrel was the most favored and prasugrel was the least favored P2Y12-I with increasing age, specifically in ≥75 years or older. This trend is consistent with a black-box warning against prasugrel use in ≥75 years or older due to increased risk of major bleeding.^16^ Whether ticagrelor will gain popularity in this age-subgroup remains to be established. As far as safety data is concerned for use of ticagrelor over clopidogrel in this age-subgroup, the PLATO participants ≥75 years or older (n=2,878, 15% of the participants) did not experience increased adverse events with ticagrelor use.^4^ However, effectiveness data on this age-subgroup reported lack of superiority of ticagrelor over clopidogrel to reduce a composite of cardiovascular death, nonfatal AMI or nonfatal stroke, HR 0.94 (0.78-1.12).^4^ These reasons may explain why clopidogrel was favored over other P2Y12-I for ≥75 years or older. We found men were 19% less likely to receive ticagrelor over prasugrel and 23% more likely to receive prasugrel over clopidogrel. We also found racial differences in the choice of P2Y12-I, with minorities less likely than Caucasians to receive ticagrelor or prasugrel over clopidogrel. Two-thirds of the cohort was receiving low-income subsidy and we did not find an association between choice of P2Y12-I and receipt of the subsidy. Unmeasured variables related to socioeconomic status might play an important role in the choice of P2Y12-I given cost differences between the drugs which might explain these findings.

Dialysis-related factors were also associated with choice of P2Y12-I in patients with ESRD. Individuals on hemodialysis versus peritoneal dialysis had 33% lower odds of receiving prasugrel over clopidogrel. In addition, for every year increase in time on dialysis, there was a 9% lower odds of receiving prasugrel over clopidogrel. Patients on chronic dialysis are already at heightened risk of major gastrointestinal bleeding.^17–19^ This risk continues to worsen with increasing dialysis vintage due to multiple reasons including development of arteriovenous malformations or because of receiving heparin during hemodialysis treatments. These complications might drive clinicians to avoid prasugrel in ESRD patients who have been on chronic dialysis for longer duration due to fear of major bleeds, specifically gastrointestinal bleeds.^4, 20^ This might also be the reason why prasugrel remains under-utilized in this patient population and the trend remained flat during the study period.

Presence of atrial fibrillation or COPD compared to absence of these comorbidities reduced the odds of receiving ticagrelor or prasugrel over clopidogrel. Ticagrelor was reported to cause dyspnea and abnormal heart beats in the PLATO trial.^4^ This might dissuade clinicians to prescribe ticagrelor in the setting of atrial fibrillation or COPD and might explain these results. Lack of association between concurrent anticoagulant use (*versus no use*) at index date with choice of P2Y12-I might be a finding by chance due to small number of patients on ticagrelor or prasugrel who were prescribed an oral anticoagulant. Recent RCTs that reported safety of dual therapy with anticoagulants and P2Y12-I were published after the study period and are unlikely to explain this trend.^21, 22^

Finally, occurrence of NSTEMI or PCI compared to absence of these events within the 6-month period prior to the index date favored ticagrelor over other P2Y12-I. In the setting of non-fatal cardiogenic shock or non-fatal cardiac arrest, there were higher odds of receiving prasugrel over clopidogrel. However, when multiple or drug-eluting coronary stents were deployed, prescribing trends favoring ticagrelor over prasugrel were not observed. Some of these choices indicate utilization of P2Y12-I prescriptions according to the FDA-approved clinical indication for P2Y12-I use in the general population. There are several differences in clinical indication for use of each P2Y12-I.^16, 23, 24^ Clopidogrel is FDA approved for acute coronary syndrome (ACS) to reduce the rate of cardiovascular death, AMI or stroke.^25^ In addition, it is also approved for use in patients with recent AMI, recent stroke or established PVD to reduce the rate of new ischemic stroke, new AMI and other vascular death.^26^ Prasugrel is FDA approved for maintenance P2Y12-I therapy in patients with acute coronary syndrome who are treated with PCI and are not at high risk of bleeding complication and who do not have a history of stroke.^27^ Ticagrelor is FDA approved for maintenance P2Y12 therapy in patients with acute coronary syndrome who are treated with PCI or medical therapy alone.^27^ Although the FDA drug label for ticagrelor does not restrict its use in patients with history of stroke, the secondary review of PLATO trial data by the FDA noted 2-5 times higher risk of stroke in the *ticagrelor* arm versus the *clopidogrel* arm.^15^ On the one hand, differences in FDA approved clinical indication for use and unclear efficacy in non-cardiac thrombotic cardiovascular events might explain choice of clopidogrel over ticagrelor or prasugrel for patients on chronic dialysis with certain comorbidities. On the other hand, some trends in our results need further research with data from recent years, for e.g., prescribing trends favoring prasugrel over clopidogrel in nonfatal cardiogenic shock or cardiac arrest, and lack of preference for ticagrelor over prasugrel in the deployment of drug-eluting stents. Patients with more severe cardiac events at the index dates might be more likely to receive prasugrel or ticagrelor over clopidogrel. Recent pharmacodynamics studies reported greater and quicker platelet inhibition with prasugrel or ticagrelor over clopidogrel in patients receiving hemodialysis.^28–31^ Due to small sample size in the ticagrelor group of the cohort, we might have failed to observe trends favoring ticagrelor over other P2Y12-I in these settings.

This study has several strengths. We describe national trends in prescribing P2Y12-I for patients with ESRD who are older and racially diverse. Because this is an understudied population with scarce efficacy and safety data of P2Y12-I use, we also describe clinical factors that may be associated with physicians’ choices when prescribing these drugs to ESRD patients. By studying new prescription users, we reduced prevalence bias in the findings. This study also has limitations. First, we may have missed some covariates or clinical risk factors related to new prescriptions for P2Y12-I, including obesity, coronary anatomy, and thrombolysis in myocardial infarction flow pre- and post-PCI. Second, we did not have information about concomitant aspirin use because it may be purchased over the counter. Patients may be prescribed P2Y12-I as monotherapy without (or instead of) aspirin, particularly considering the bleeding risks associated with dual antiplatelet therapy in this patient population. Without aspirin data, trends reported in the manuscript may not be complete. Despite lack of aspirin data, our results provide understanding of current clinical practice and utilization of P2Y12-I in an understudied and high-risk patient population. Third, general limitations of pharmacoepidemiological studies using administrative claims data may also exist including accuracy in coding, lack of direct comparison of the study population with P2Y12-I use in patients without ESRD, lack of socioeconomic data, and lack of outcomes data related to either future thrombotic events or bleeding while on P2Y12-I treatment.

In summary, clopidogrel remained the most common P2Y12-I prescribed to patients on chronic dialysis between 2011 and 2014. During the study period, ticagrelor and prasugrel remained underutilized, prasugrel utilization plateaued and ticagrelor utilization was on the rise. Age and racial differences determined choice of P2Y12-I prescriptions: clopidogrel the most favored and prasugrel the least favored with older age; and, minorities less likely to receive ticagrelor over others P2Y12-I. Among dialysis-related factors, patients who were on dialysis for longer duration or receiving hemodialysis versus peritoneal dialysis were less likely to be prescribed prasugrel over clopidogrel. Among non-cardiac comorbidities or cardiac events during the 6-month period prior to the index date, prescribing patterns primarily indicate utilization of P2Y12-I prescriptions according to the FDA-approved clinical indication for their use in the general population. Comparative effectiveness studies involving ESRD patients are needed to prove that ticagrelor and prasugrel are just as safe and effective as clopidogrel before clinicians can make informed decisions for choice of P2Y12-I in this patient population.

## Electronic supplementary material

ESM 1 (DOCX 26 kb)
