# Supplementary material for: Trends for and Clinical Factors Associated with Choice of Oral P2Y12 Inhibitors for Patients on Chronic Dialysis
Source: Cardiovasc Drugs Ther. 2019 Nov 15;33(5):511–21. doi: 10.1007/s10557-019-06913-w (PMC6904390; doi:10.1007/s10557-019-06913-w)
Supplement: Supplementary file 1 — (DOCX 26 kb) [file 10557_2019_6913_MOESM1_ESM.docx]

**Supplementary Table 1. Codes Used to Identify Baseline Characteristics of the Cohort**

| **Baseline Characteristic** | **USRDS file** | **Field name** | **Field values** |
| --- | --- | --- | --- |
| Age | Patients | first_se, born |  |
| Gender | Patients | sex |  |
| Ethnicity | Patients | hispanic |  |
| Race | Patients | Race |  |
| Residence location | Patients | County |  |
|  | USDA ERS: Rural-urban-continuum-codes | FIPS_county, rucc_2013 |  |
| Low-income subsidy | pdenrol2011, pdenrol2012, pdenrol2013, pdenrol2014 | cst_shr_grp_cd_201101- cst_shr_grp_cd_201412 | 01-08, 10-13 |
| Medicare Advantage plan enrollment | pdenrol2011, pdenrol2012, pdenrol2013, pdenrol2014 | ptd_cntrct_201101 - ptd_cntrct_201412 | H, R |
| Etiology of ESRD | Patients | Pdis |  |
| Treatment modality | RxHist | rxgrp1-rxgrp137, used field associated with index prescription | Hemodialysis: 1,2,3  Peritoneal: 5,7,9 |
| Anticoagulant use | pde2011, pde2012, pde2013, pde2014 | Gnn |  |
| Dialysis vintage | Patients | first_se, date of index P2Y12 |  |
| History of smoking | medevid | smoke, como_tobac | Y |
| Hypertension | medevid | como_htn, hyper | Y |
|  | medevid | pdis | 401, 403 |
| Diabetes | medevid | diabins, diabprim, como_dm_ret, como_dm_oral, como_dm_nomeds | Y |
|  | medevid | pdis | 250, 3572, 3620, 36641 |
|  | Hosp2010_on | Hsdiag1-Hsdiag26 | ICD-9: 250, 3572, 3620, 36641 |
|  | Physician/Supplier claims | diag | ICD-9: 250, 357.2, 362.0x, 366.41 |

**Codes Used to Define Comorbidities of the Cohort Using Physician/Supplier Claims, Institutional Claims and CMS Form 2728**

| **Baseline Characteristic** | **DRG codes** | **ICD-9-CM Diagnoses** | **ICD-9-CM procedures** | **CPT, HCPC Procedures** |  |  |  |  |  |
| --- | --- | --- | --- | --- | --- | --- | --- | --- | --- |
| AMI | 222, 223**,** 280-285 | STEMI: 410.0, 410.6, 410.8 NSTEMI: 410.7 AMI 410, 410.X0, 410.X1 |  |  |  |  |  |  |  |
| Cardiogenic shock or cardiac arrest | 222, 291-293, 296-298 | Cshock 785.51, Carrest 427.5 |  |  |  |  |  |  |  |
| Acute coronary syndrome |  | 411, 410.x except 410.x2 |  |  |  |  |  |  |  |
| CABG | 231-236 |  | 36.1x | CPT 3350-33523, 33533-33536 |  |  |  |  |  |
| PCI performed | 216-218, 223-225, 246-251 |  | 00.45, 00.46, 00.47, 00.48, 00.55, 36.06, 36.07, 39.90 | CPT 92928, 92929, 92933, 92934, 92937, 92938, 92941, 92943, 92944, 92975, 92977, 92980, 92981, 92982, 92984, 92995, 92996,  HCPCS C9600-C9608, G0290, G0291 |  |  |  |  |  |
| Coronary stents deployed | 246-249 |  | 00.45, 00.46, 00.47, 00.48 | CPT 92928, 92929, 92933, 92934, 92980, 92981 HCPCS G0290 G0291 C9600 C9601 C9602 C9603 |  |  |  |  |  |
| Multiple coronary stents deployed | 246, 248 |  | 00.46, 00.47, 00.48 | CPT 92929, 92934, 92944, 92981 HCPCS C9601, C9603, G0291 |  |  |  |  |  |
| Drug eluting stent deployed | 246, 247 |  | 36.07, 00.55 | HCPC G0290 G0291 C9600 C9601 C9602 C9603 |  |  |  |  |  |
| Bare metal stent deployed | 248, 249 |  | 36.06, 39.90 | CPT 92928, 92929, 92933, 92934, 92980, 92981 |  |  |  |  |  |
| Atrial Fibrillation |  | 427.31 |  |  |  |  |  |  |  |
| Congestive Heart Failure |  | 398.91, 402.01, 402.11, 402.91, 404.01, 404.03, 404.11, 404.13, 404.91, 404.93, 425.4-425.9, 428.x |  |  |  |  |  |  |  |
|  | Form 2728 como_chf=Y | |  |  |  |  |  |  |  |
| Abnormal Stress Test |  | 794.39 |  |  |  |  |  |  |  |
| Peripheral vascular disease | 239-241, 255-257, 299-301 | 440-444 |  |  |  |  |  |  |  |
|  | Form 2728 como_pvd=Y | |  |  |  |  |  |  |  |
| Amputations | 239-241, 255-257, 474-476 |  | 84.11 |  |  |  |  |  |  |
| Ischemic stroke |  | 433.x1, 434.x1, 436 |  |  |  |  |  |  |  |
| Intracranial hemmorrhage |  | 430-432 |  |  |  |  |  |  |  |
| Chronic obstructive pulmonary disease |  | 491-494, 496, 510 |  |  |  |  |  |  |  |
|  | Form 2728 como_copd=Y | |  |  |  |  |  |  |  |
| Modified Liu Index _1-180_ |  | | | |  |  |  |  |  |
| Atherosclerotic heart disease |  | 410-414, V45.81, V45.82 |  |  |  |  |  |  |  |
| Congestive heart failure |  | 398.91, 422, 425, 428, 402.x1, 404.x1, 404.x3, V42.1 |  |  |  |  |  |  |  |
| Cerbrovascular accident / transient ischemic attack |  | 430-438 |  |  |  |  |  |  |  |
| Peripheral vascular disease |  | 440-444, 447, 451-453, 557 |  |  |  |  |  |  |  |
| Other cardiac |  | 420-421, 423-424,429, 785.0-785.3, V42.2, V43.3 |  |  |  |  |  |  |  |
| Chronic obstructive pulmonary disease |  | 491-494, 496, 510 |  |  |  |  |  |  |  |
| Gastrointestinal bleeding |  | 456.0-456.2, 530.7, 531-534, 569.84, 569.85, 578 |  |  |  | 430-438 |  |  |  |
| Liver disease |  | 570, 571, 572.1, 572.4, 573.1-573.3, V42.7 |  |  |  | 440-444, 447, 451-453, 557 |  |  |  |
| Dysrhythmia |  | 426-427, V45.0, V53.3 |  |  |  | 491-494, 496, 510 |  |  |  |
| Cancer |  | 140-172, 174-208, 230-231, 233-234 |  |  |  |  |  |  |  |
| Diabetes |  | 250, 357.2, 362.0x, 366.41 |  |  |  |  |  |  |  |
